# A Large-Scan-Range Electrothermal Micromirror Integrated with Thermal Convection-Based Position Sensors

**DOI:** 10.3390/mi15081017

**Published:** 2024-08-08

**Authors:** Anrun Ren, Yingtao Ding, Hengzhang Yang, Teng Pan, Ziyue Zhang, Huikai Xie

**Affiliations:** 1 School of Integrated Circuits and Electronics, Beijing Institute of Technology, Beijing 100081, China; changfengrens@163.com (A.R.); ytd@bit.edu.cn (Y.D.); hengzhangyang@bit.edu.cn (H.Y.); tengpan@bit.edu.cn (T.P.); zyzhang@bit.edu.cn (Z.Z.); 2 Engineering Research Center of Integrated Acousto-Opto-Electronic Microsystems, Ministry of Education of China, Beijing 100081, China; 3 Chongqing Institute of Microelectronics and Microsystems, Beijing Institute of Technology, Chongqing 400030, China

**Keywords:** electrothermal micromirror, large scan range, mirror plate position sensing, thermal convection

## Abstract

This paper presents the design, simulation, fabrication, and characterization of a novel large-scan-range electrothermal micromirror integrated with a pair of position sensors. Note that the micromirror and the sensors can be manufactured within a single MEMS process flow. Thanks to the precise control of the fabrication of the grid-based large-size Al/SiO_2_ bimorph actuators, the maximum piston displacement and optical scan angle of the micromirror reach 370 μm and 36° at only 6 Vdc, respectively. Furthermore, the working principle of the sensors is deeply investigated, where the motion of the micromirror is reflected by monitoring the temperature variation-induced resistance change of the thermistors on the substrate during the synchronous movement of the mirror plate and the heaters. The results show that the full-range motion of the micromirror can be recognized by the sensors with sensitivities of 0.3 mV/μm in the piston displacement sensing and 2.1 mV/° in the tip-tilt sensing, respectively. The demonstrated large-scan-range micromirror that can be monitored by position sensors has a promising prospect for the MEMS Fourier transform spectrometers (FTS) systems.

## 1. Introduction

Electrothermal MEMS mirrors can be widely applied in optical bioimaging [1,2], 3D sensing [3,4,5], and miniature FTS [6], attributed to their large linear scan range and low driving voltage. The actuation of electrothermal micromirrors is provided by thermal bimorph beams. A thermal bimorph is made of two materials with significantly different thermal expansion coefficients. A metallic resistor is typically embedded into the bimorph to generate Joule heating, thus changing the temperature of the bimorph, which in turn leads to a curvature change of the bimorph. So, a thermal bimorph can be regarded as an electrical-thermal-mechanical coupling system. Any vibrations, humidity and temperature variations, or airflow disturbances in the working environment, will change the response of every bimorph actuator. Therefore, in order to achieve stable operation of electrothermal MEMS micromirrors, it is important to have the accurate position (vertical displacement and optical scan angle) of the mirror plate in real time. Most applications need angular scanning of optical beams, such as in endoscopic OCT [2] or MEMS LiDAR [7]. Some other applications require phase modulation or piston motion, such as in optical phased arrays [8] or FTS [9,10,11]. In MEMS FTS, the mirror plate needs to have a large piston displacement (hundreds of micrometers) with minimal tilting to achieve high spectral resolution. Thus, real-time monitoring and closed-loop control of the mirror plate position are necessary, demanding position-sensing components to be integrated.

**Figure 1:**
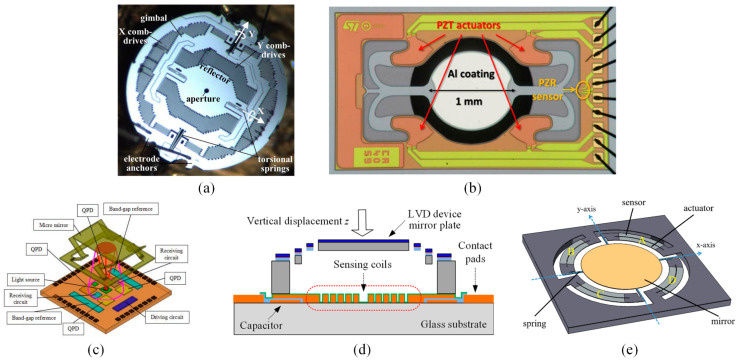
Schematic diagrams of different position sensing methods: (**a**) capacitive sensing [13]; (**b**) piezoresistive sensing [19]; (**c**) optical sensing [21]; (**d**) inductive eddy current sensing [26]; (**e**) piezoelectric sensing [29].

Different position-sensing methods of the mirror plate are illustrated in Figure 1. Capacitive sensing (Figure 1a) based on comb fingers [12,13] or parallel-plate electrodes [14] can provide high resolution [15] in dynamic measurements, but the sensing capacitances are typically very small and thus it is quite challenging to handle parasitic capacitances. Piezoresistive sensing (Figure 1b) is commonly used for piezoelectric micromirrors [16,17,18,19] and shows a good performance in quasi-static and dynamic measurements, but it has a serious nonlinear issue at large displacement and is temperature-sensitive, making it difficult to be integrated with electrothermal micromirrors. Optical sensing (Figure 1c) can provide suitable resolution and satisfactory sensing range [20,21,22] in both quasi-static and dynamic measurements but requires a dedicated assembly of extra off-chip components [22]. For example, two position sensing detectors were used to achieve 2D tilt-angle sensing of an electrothermal micromirror [23]; this method is not suitable for applications in narrow spaces. Inductive eddy current sensing (Figure 1d) is also developed to detect the mirror plate position and achieve nanometer resolution [24,25,26,27] in dynamic sensing measurements, but it requires off-chip bulky coils for large sensing range and high resolution, which is unfavorable for miniaturization. Piezoelectric sensing (Figure 1e) can also be used for position detection in piezoelectric micromirrors [28,29] and show a good performance in dynamic measurements. However, the incorporation of the piezoelectric effect into electrothermally-actuated bimorph structures is challenging as the properties of piezoelectric materials are largely affected by temperature change.

In this work, the goal is to figure out a method that can realize position sensing of an electrothermal micromirror mirror plate that can move with large vertical displacement (e.g., 300 μm) and optical scan angle of tip-tilt (e.g., 20°). For that, we have developed a novel position sensor utilizing the temperature distribution based on thermal convection, which includes one heater and one thermistor. The heater is connected to the mirror plate, which moves together with the mirror plate and creates a temperature distribution. The thermistor is used to detect the temperature change caused by the position variation of the mirror plate, where the temperature change around the thermistor will cause its resistance change. Then, the resistance change is converted into an output voltage through a Wheatstone bridge circuit. Thus, the detection of the mirror plate position is achieved. Since no extra component is involved, the device structure is simple. And the fabrication process of the position sensor is completely compatible with that of the electrothermal micromirror. The preliminary results were reported in IEEE Transducers 2023 [30]. In this paper, the LSF bimorph actuators and position sensors of the proposed micromirror have been optimized to enhance the scan range and sensing range. In addition, more detailed simulation and test results have been added.

In the following sections, the working principle of the position sensor is first introduced in Section 2, and the design of the micromirror integrated with position sensors and the simulation results of the sensor are discussed in Section 3. Then, in Section 4, the fabrication process is described. After that, the experimental characterization results of the fabricated micromirror and the integrated position sensors are demonstrated in Section 5.

## 2. Working Principle of the Position Sensor

**Figure 2:**
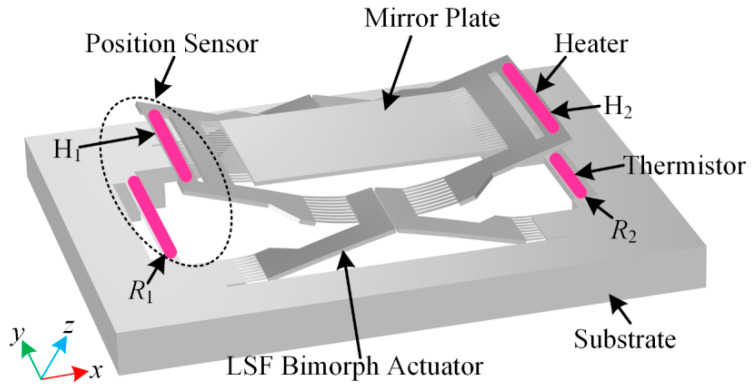
Schematic illustration of the electrothermal micromirror integrated with thermal convection-based position sensors.

Figure 2 shows a schematic illustration of the proposed electrothermal micromirror device integrated with two thermal convection-based position sensors located oppositely at the two sides of the mirror. The mirror plate is supported by lateral-shift-free (LSF) bimorph actuators which can provide a large scan range for the mirror plate. Each position sensor consists of two resistors serving as the heater and the thermistor, respectively. Two heaters (H_1_ and H_2_) are located at the mirror plate, and two thermistors (*R*_1_ and *R*_2_) are on the substrate underneath the heaters.

**Figure 3:**
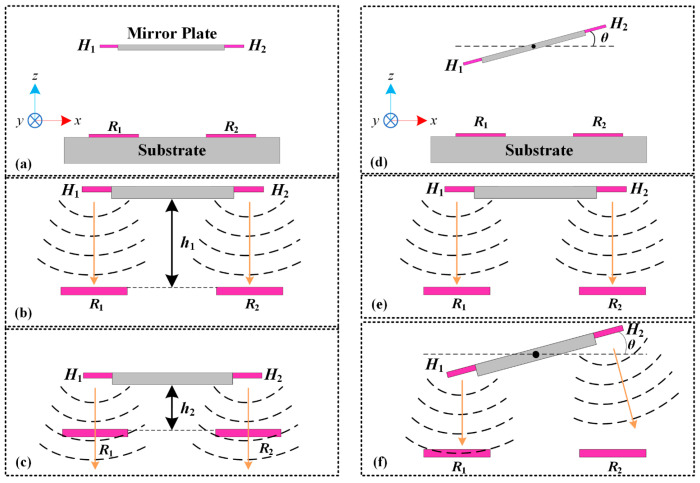
Working principles of the position sensors; (**a**–**c**) piston sensing; (**d**–**f**) tip-tilt sensing.

The working principles of the position sensors for both the piston sensing and the tip-tilt sensing are shown in Figure 3. For the electrothermal micromirrors discussed in this work, the highest temperature in the bimorph actuators does not exceed 300 °C. Under this condition, heat transfer through thermal radiation is negligible compared to heat transfer by thermal conduction and thermal convection [31,32]. According to the thermal convection theory, the temperature distribution around the thermistors changes with the relative position variations of the heaters, which reflect the movement of the mirror plate. For the piston sensing in Figure 3a, the initial temperature distribution in the *x*-*z* plane is shown in Figure 3b, where the initial vertical distance between the mirror plate and the thermistors is *h*_1_. With the mirror plate moving downwards, the vertical distance between the mirror plate and the thermistors reduces to *h*_2_ in Figure 3c. As the heaters are closer to the thermistors, the temperatures of the thermistors are risen. The temperature variation can be expressed as

Δ*T*_h_ = *T*(*R*_1_) − *T*_0_, (1)

where Δ*T*_h_ denotes the temperature difference induced by the vertical displacement of the mirror plate. *T*(*R*_1_) is the temperature of the thermistor *R*_1_, and *T*_0_ is the environment temperature.

Similarly, for the tip-tilt sensing in Figure 3d, when the mirror plate parallels the substrate surface, the temperature of *R*_1_ is equal to that of *R*_2_ (Figure 3e). As shown in Figure 3f, with the mirror plate tilting at the angle of *θ*, the heater H_1_ moves closer to *R*_1_ while the heater H_2_ moves further to *R*_2_; thus, the temperature of *R*_1_ is higher than that of *R*_2_. The temperature changes can be expressed as

Δ*T*_θ_ = *T*(*R*_1_) − *T*(*R*_2_), (2)

where Δ*T*_θ_ denotes the temperature difference induced by the tip-tilt of the mirror plate.

**Figure 4:**
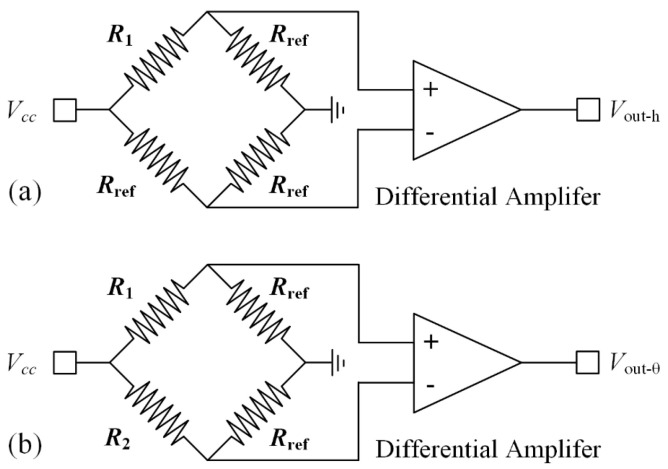
Schematic diagrams of the Wheatstone bridge circuits: (**a**) piston sensing circuit. (**b**) tip-tilt sensing circuit.

The temperature differences of the thermistors can be converted to the voltage outputs of corresponding Wheatstone bridge circuits, as illustrated in Figure 4. The output voltages can be expressed as

*V*_out-h_ = AαΔ*T*_h_·*V_cc_*, (3)

*V*_out-θ_ = AαΔ*T*_θ_·*V_cc_*, (4)

where A, α, and *V_cc_* are the constant of the circuit, the temperature coefficient of resistance (TCR) of the thermistors, and the supply voltage of the bridge circuit, respectively. *R*_ref_ is a reference resistor used to balance the circuit. Since the thermistor *R*_1_ is used in both piston and tip-tilt sensing circuits, different modulation frequencies are applied to the bias voltages of these two circuits to minimize the crosstalk between the motions.

## 3. Structure Design of the Device and Simulation of the Sensing Performance

**Figure 5:**
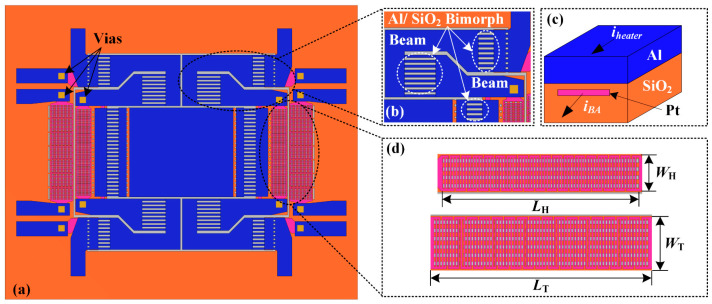
Schematic diagrams of the proposed device and the key components: (**a**) top view of the device; (**b**) enlarged view of the bimorph actuator; (**c**) 3D structure of the bimorph; (**d**) enlarged view of the position sensor and the critical structural parameters.

Figure 5 shows the structural schematics of the designed micromirror integrated with position sensors. The bimorph actuator is composed of three grid-based large-size Al/SiO_2_ bimorph structures connected by two beams, as shown in Figure 5b. The Pt layer is embedded in the bimorph structures for uniform and efficient heating, as shown in Figure 5c. Note that the current for the heater (*i_heater_*) is applied through the Al layer. The current for the bimorph actuator (*i_BA_*) is applied through the Pt layer embedded in the SiO_2_ layer. The structural parameters of the device are listed in Table 1.

**Figure 6:**
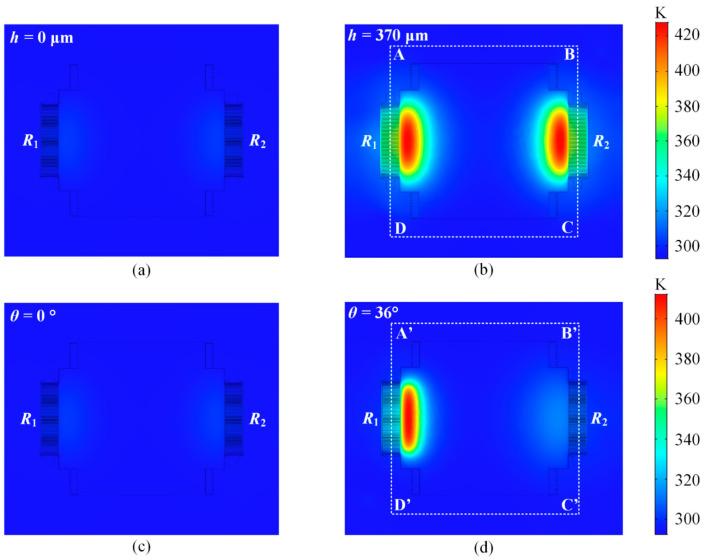
Simulated temperature distributions of the position sensors during working at *T*_0_ = 293 K. (**a**,**b**) Piston sensing. (**c**,**d**) Tip-tilt sensing.

**Figure 7:**
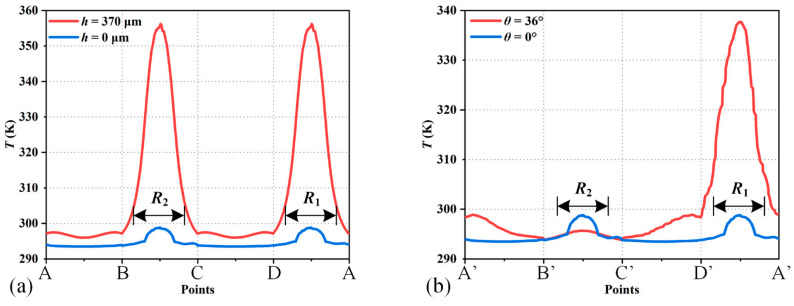
Extracted temperature curves along the indicated lines in Figure 6: (**a**) piston sensing; (**b**) tip-tilt sensing.

The performance of the position sensors is studied by FEA (finite element analysis) using COMSOL Multiphysics software 5.5. Figure 6 shows the simulation results of the temperature distribution near the thermistors while the position sensors are working at *T*_0_ = 293 K. As shown in Figure 6a,b, *R*_1,_ and *R*_2_ are in the same temperature gradient when the mirror plate vertically displaces downwards for 370 μm, where the temperature increases compared with the initial temperature. In Figure 6d, the temperature of *R*_1_ is higher than that of *R*_2_ when the mirror plate tilts at an optical angle of 36°. Figure 7 plots the temperature curves along the A-B-C-D-A direction and the A’-B’-C’-D’-A’ direction. According to the different temperature distributions, the position of the mirror plate can be detected.

**Figure 8:**
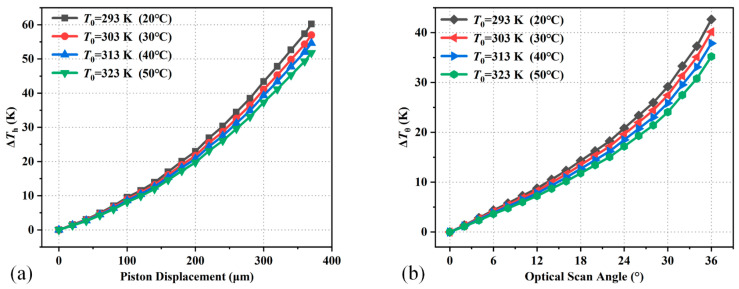
Temperature variations of the thermistors under different environment temperatures: (**a**) piston displacements; (**b**) optical scan angles.

Furthermore, the temperature variations of the thermistors in different environment temperatures (*T*_0_) are also simulated. Figure 8 shows the simulated temperature variations of the thermistors with different piston displacements or optical scan angles of the mirror plate under different environment temperatures. In Figure 8a, it can be seen that Δ*T*_h_ increases with the piston displacement of the mirror plate and decreases with *T*_0_. In Figure 8b, Δ*T*_θ_ also increases with the optical scan angle of the mirror plate and decreases with *T*_0_. The simulated sensing ranges of piston displacement and optical scan angle are 370 μm and 36°, respectively. Table 2 lists the theoretical sensitivity of position sensors at different environment temperatures after linear fitting. The sensitivities of piston sensing and tip-tilt sensing decrease while T0 increases. In piston sensing, the estimated sensitivity decreases by about 1 mK/μm for every 1 K increase in the environment temperature.

## 4. Fabrication Process

**Figure 9:**
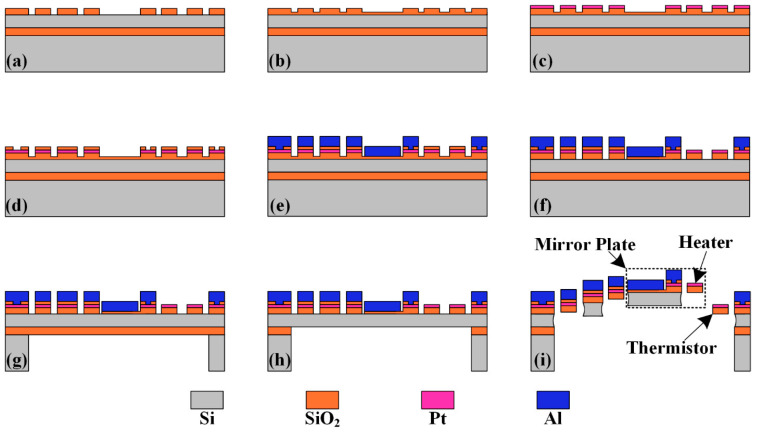
Fabrication process of the large-scan-range electrothermal micromirror integrated with position sensors. (**a**) PECVD oxide deposition and etching. (**b**) PECVD oxide deposition. (**c**) Pt sputtering and lift-off. (**d**) PECVD oxide deposition and etching. (**e**) Al sputtering and etching. (**f**) Oxide etching. (**g**) Backside Si etching. (**h**) Backside box layer etching. (**i**) Frontside Si isotropic etching.

The fabrication process of the proposed micromirror integrated with position sensors is shown in Figure 9. A simple surface- and bulk-combined micromachining process based on an SOI wafer is used, and the handle layer and device layer thicknesses of the wafer are 425 μm and 25 μm, respectively. The thickness of the buried oxide (BOX) layer is 1 μm, which will be used as the etch-stop layer of the back-side deep reactive ion etch (DRIE) process. First, a 1 μm-thick SiO_2_ layer is deposited through plasma-enhanced chemical vapor deposition (PECVD) and patterned through the buffered oxide etch (BOE) process in Figure 9a. In Figure 9b, a 0.1 μm-thick SiO_2_ layer is then deposited by PECVD. In Figure 9c, a 0.15 μm-thick Pt layer is sputtered and patterned through lift-off. A 0.1 μm-thick SiO_2_ layer is then deposited by PECVD and some vias are formed by the reactive ion etch (RIE) process in Figure 9d. After that, a 1 μm-thick Al layer is deposited and patterned by RIE in Figure 9e. The 0.2 μm-thick SiO_2_ layer is then removed by RIE to form the bimorph structure in Figure 9f. Next, a Si cavity is etched down to the BOX layer from the backside of the SOI wafer by DRIE in Figure 9g, followed by the removal of the 1 μm-thick BOX layer by RIE in Figure 9h. Finally, the microstructures are released from the frontside by isotropic etching using xenon difluoride (XeF_2_) in Figure 9i.

**Figure 10:**
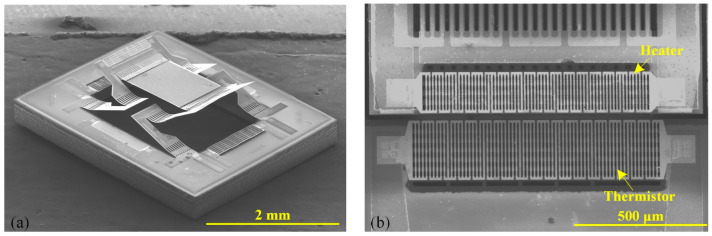
Images of the fabricated electrothermal micromirror integrated with position sensors: (**a**) the fabricated device after release; (**b**) the close-up view of the heater and the thermistor.

Figure 10 shows the SEM images of the fabricated electrothermal micromirror, where the mirror plate size is 1 mm × 0.9 mm. The mirror plate is supported by two LSF bimorph actuators and elevated by 370 μm.

## 5. Experimental Characterizations

### 5.1. Quasi-Static and Dynamic Response of the Micromirror

**Figure 11:**
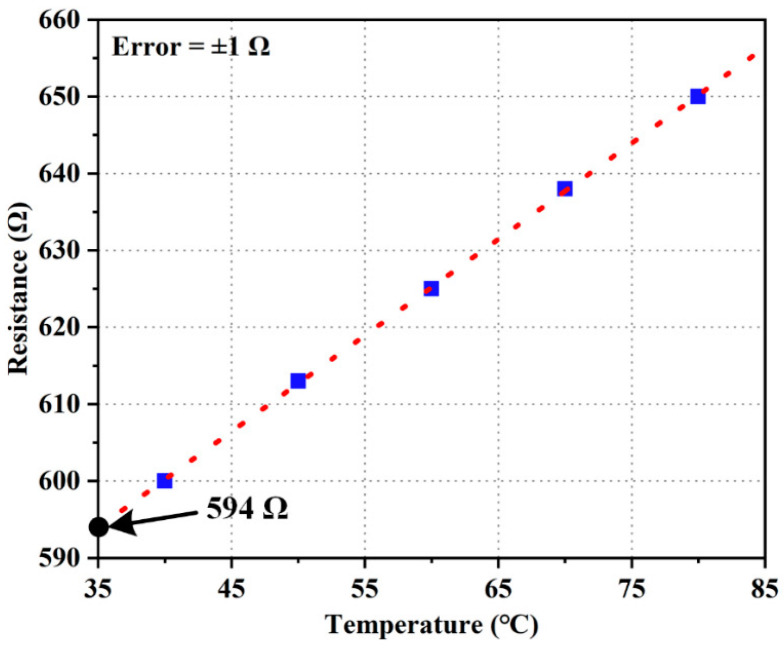
Measured resistance-temperature curve for the Pt resistor in the bimorph.

The TCR of a thin-film Pt resistor is usually different from that of bulk Pt and depends on the deposition process. Hence, the measurement of the TCR of the Pt resistor is necessary. It is measured by placing the device into a thermal chamber with a temperature accuracy of ±0.1 °C. Figure 11 plots the measured resistances under different temperatures, and the TCR is calculated to be 0.0023/°C.

**Figure 12:**
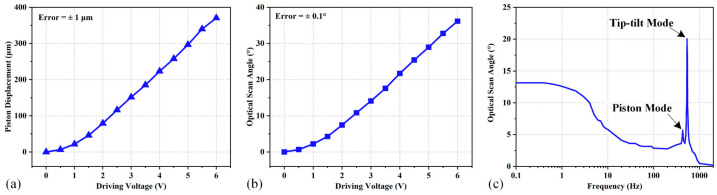
The quasi-static and dynamic responses of the micromirror: (**a**) piston displacement versus driving voltage; (**b**) optical angle versus driving voltage; (**c**) frequency response.

The piston displacement and optical scan angle are measured by a position-sensitive detector (PSD). When a laser beam points at a working micromirror, the PSD records the position of the laser spot reflected from the mirror plate, and then the scan range can be deduced. The measured piston displacement of the mirror plate versus the driving voltage is plotted in Figure 12a, where the displacement reaches 370 μm at only 6 Vdc and good linearity is observed for the displacement range from 40 to 370 μm, where the linearity is 1.2%. The driving current and driving power of the two actuators at 6 Vdc are 14.2 mA and 85.2 mW, respectively. Figure 12b shows the optical scan angle versus the driving voltage applied to one of the actuators, where the optical scan angle reaches 36° at 6 Vdc, with the corresponding driving current and driving power at 7.1 mA and 42.8 mW, respectively. The linearity in the range from 4.2° to 36° can reach 1.5%. The micromirror shows lager-scan-range and good linearity in piston displacement and optical scan angle, which is very popular and desirable in FTS applications. The frequency response of the micromirror is measured by applying a sinusoidal voltage varying between 0 and 3 V to one LSF actuator of the micromirror. The response curve in the frequency range of 0.1 to 2000 Hz is plotted in Figure 12c, where the first-order resonance is 432 Hz and the second-order resonance is 536 Hz, respectively. The dynamic response of the electrothermal micromirror is represented as a first-order thermal system connected with a second-order spring-mass system. A more detailed analysis can be found in the reference [33]. As shown in Figure 12c, the 3 dB thermal cutoff frequency (*f_cutoff_*) is only 5 Hz, Hence, the optical scan angle decreases significantly starting from 5 Hz all way to 100 Hz, after which the optical scan angle increases with increasing frequency due to the mechanical resonance.

### 5.2. Quasi-Static Characterizations of the Position Sensor

**Figure 13:**
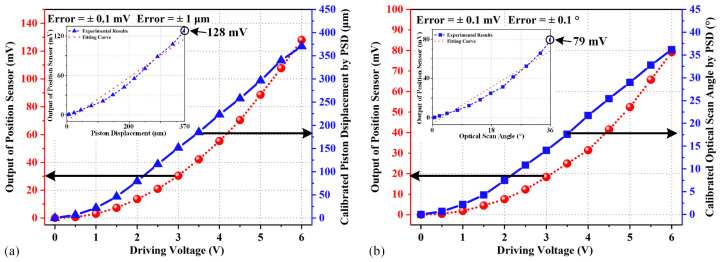
Quasi-static measurement results: (**a**) the output of the position sensor and calibrated piston displacement versus the driving voltage of two actuators; (**b**) the output of the position sensor and calibrated optical scan range versus the driving voltage of one actuator.

In the quasi-static measurements, the outputs of the position sensor are recorded while the driving voltage is applied to actuators. A PSD is also used to acquire the accurate position of the mirror plate, which is used to calibrate the output of the position sensor. The test results are shown in Figure 13. For the piston sensing, when the displacement of the mirror plate reaches 370 μm, the position sensor output is 128 mV in Figure 13a. For the tip-tilt sensing, when the optical scan angle of the mirror plate reaches 36°, the position sensor output is 79 mV in Figure 13b. Note that the PSD is employed to calibrate the optical scan angles of the mirror plate under a range of driving voltage, as shown in Figure 12b. In Figure 13, the vertical axis on the left is the output voltage of the thermistor while the vertical axis on the right is the optical scan angle extracted from the PSD calibration given in Figure 12b. It can be seen that the sensing ranges of the sensor can indeed cover the full motion of the mirror plate in both piston displacement and optical scan angle. And the linearities are 9.3% F.S and 4.9% F.S with sensitivities of 0.3 mV/μm and 2.1 mV/°, respectively. The large scan range and sensing range of the micromirror can satisfy the requirements for FTS systems. Since the reflectivity of the mirror surface is only about 90%, the laser power absorbed will cause the temperature of the mirror plate to increase considerably if the laser power is high such as in the case of long-distance LiDAR. Thus, the outputs of the position sensor need to be re-calibrated to reduce measurement errors if high-power lasers are involved.

## 6. Conclusions

The fundamental mechanism analysis, design, simulation, fabrication, and characterizations of a large scan range electrothermal micromirror integrated with position sensors are discussed in this paper. The proposed method for the mirror plate position detection is an on-chip integration solution and does not need to assemble off-chip components. The fabrication process of the sensor is compatible with the micromirror. Measurement results of the micromirror show that the displacement and optical scan angle in quasi-static mode can reach 370 μm and 36° at only 6 Vdc voltage, where the linearities of piston displacement range from 40 μm to 370 μm and optical scan range from 4.2° to 36° can reach 1.2% and 1.5%, respectively. Such a sensing range of the sensor can fully cover the motion range of the micromirror. Furthermore, the piston displacement sensing and optical scan angle of the tip-tilt sensing measurements exhibit linearities of 9.3% F.S and 4.9% F.S, and the sensitivities are 0.3 mV/μm and 2.1 mV/°, respectively. Future efforts will be on the optimization of the sensor and micromirror design to increase the sensitivity and motion dimension. The heat transfer modeling will also be studied.

## Figures and Tables

**Table 1:** Structural parameters of the designed device.

Parameter	Value
Device footprint	3.6 mm × 3 mm
Mirror plate size	1 mm × 0.9 mm
Bimorph lengths	120 μm/240 μm/120 μm
Bimorph width	20 μm
Beam length	720 μm
Beam width	65 μm
Heater length	880 μm
Heater width	150 μm
Thermistor length	960 μm
Thermistor width	230 μm
Thickness of Al	1 μm
Thickness of SiO_2_	1 μm
Thickness of Pt	0.15 μm
Width of Pt in bimorph	15 μm
Width of Pt in sensor	8 μm

**Table 2:** Theoretical sensitivities of position sensors at different environment temperatures.

	*T*_0_ = 293 K	*T*_0_ = 303 K	*T*_0_ = 313 K	*T*_0_ = 323 K
Piston sensing sensitivities	0.17 K/μm	0.16 K/μm	0.15 K/μm	0.14 K/μm
Tip-tilt sensing sensitivities	1.09 K/°	1.04 K/μm	0.97 K/μm	0.91 K/μm

## Data Availability

Data are available from the authors on request.
